# Chitosan-Based Nanogels in Modern Drug Delivery: Focus on Protein and Gene Applications

**DOI:** 10.3390/gels11090735

**Published:** 2025-09-12

**Authors:** Muhammet Davut Arpa, Fatma Julide Akbuğa

**Affiliations:** Department of Pharmaceutical Technology, School of Pharmacy, Istanbul Medipol University, 34815 İstanbul, Türkiye; mdarpa@medipol.edu.tr

**Keywords:** chitosan, nanogel, protein delivery, nucleic acid, gene delivery

## Abstract

Nanogels have attracted significant attention in recent years due to their high biocompatibility, controlled release capacity, sensitivity to environmental stimuli, and targeted transport characteristics as drug delivery systems. Chitosan, a natural polysaccharide, is a biopolymer widely used in nanogel formulations due to its positively charged structure, biodegradability, and modifiable functional groups. In this review, the therapeutic applications of chitosan-based nanogels are discussed thoroughly, especially emphasizing in the areas of protein/peptide, antigen, and gene transport. Production methods, chemical modification strategies, transport mechanisms to target cells, and the biological activities of these systems have been evaluated. Chitosan nanogels are promising carrier systems in wide range of areas, including gene therapy, immunotherapy, and the delivery of biological agents, owing to their significant characteristics such as intracellular targeting, endosomal escape, and sustained release. Further studies might enable the translation of these systems into clinical applications.

## 1. Introduction

The drug delivery systems, particularly the nanogels developed from natural polymers such as chitosan, have garnered significant attention by the rise in nanotechnology in the pharmaceutical field.

Nanogels, which are formed by cross-linking polymeric particles (with a 3D structure), are rapidly developing and widely researched drug forms in pharmaceutical technology due to their stable structures, biocompatibilities, large specific surface areas, and high water contents. In their structures, nanogels integrate the favorable characteristics of nanocarriers and hydrogels, which allow the development of nanoforms with wide range of desirable features. For example, nanogels integrate the advantageous features of nanocarriers, such as enhanced drug-loading efficiency and chemical robustness, with the dynamic swelling behavior and responsiveness of hydrogels to external stimuli [1,2,3,4,5,6].

**Figure 1:**
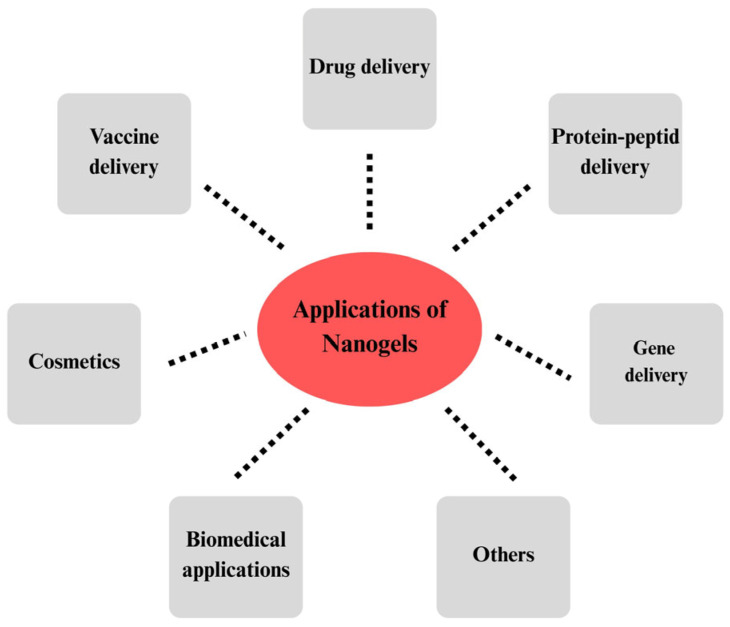
Applications of nanogels in pharmaceutical, biomedical, and cosmetic fields.

Nanogels are much preferred compared to hydrogels since they are nano-sized, have larger surface areas, remain in circulation for a longer time, and have high drug-targeting characteristics [7]. They are used in various areas such as drug, vaccine, protein, or gene delivery, biomedical applications, cancer and wound treatments, and cosmetics [7,8,9] (Figure 1).

In 1999, Vinogradov et al. were the first to name a new system prepared with polyethyleneimine and carbonyl diimidazole-activated PEG as NanoGel™ [10]. Nanogels are nano-sized, flexible, and versatile biomaterials. They are prepared from mixtures of natural, synthetic, and hybrid (i.e., natural-synthetic) polymers. Synthetic polymers are added to natural polymers to increase their effectiveness. For example, PEG increases the colloidal stability of nanosuspension-based nanogels through its known steric stabilization effect, and dendrimers can encapsulate small molecules and even nanoparticles with their tree-like nanosized structures [5].

**Figure 2:**
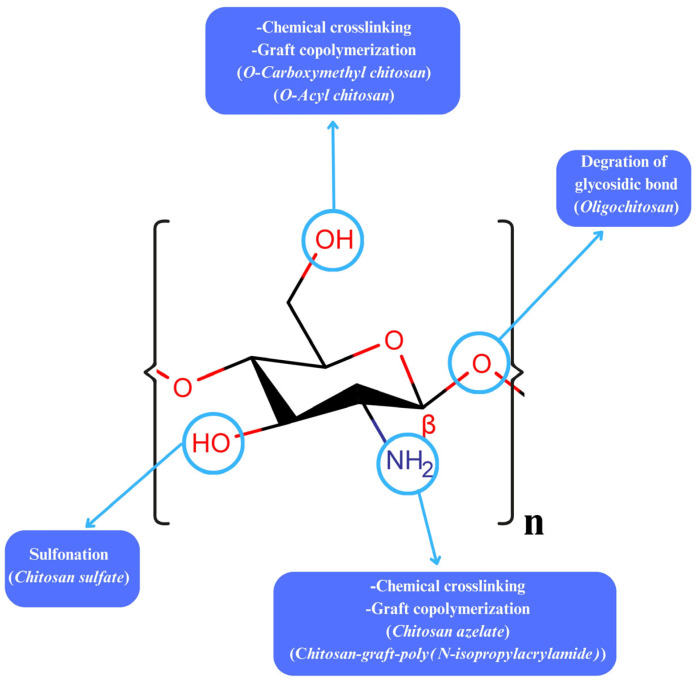
A glucosamine unit of the chitosan chain and some chemical modifications on its functional groups. This schematic illustrates the basic structure of chitosan, highlighting the –NH_2_ and –OH functional groups that enable chemical modification. These reactive sites are commonly used for crosslinking, conjugation with targeting ligands, and formation of derivatives such as carboxymethyl chitosan or thiolated chitosan. Such modifications significantly enhance chitosan’s solubility, stability, and binding capacity for proteins, genes, and other therapeutic molecules in nanogel-based drug delivery systems.

Nanogels are categorized based on their compositions and structures, abilities to respond to stimuli (e.g., pH, temperature), methods of synthesis, and routes of application [11,12]. Natural polymers frequently incorporated into nanogel systems include polysaccharides and proteins due to their biocompatibility and functional versatility [13,14]. Among these, chitosan has attracted significant research interest as a representative polysaccharide. Its cationic properties have made chitosan particularly attractive in nanogel preparation [15]. Chitosan (Figure 2) is an ideal polysaccharide that is biocompatible and biodegradable. More importantly, it has low immunogenicity and toxicity, which altogether make it essential in drug delivery systems [16,17]. Additionally, chitosan has antimicrobial, hypocholesterolemic, antioxidant, and anti-inflammatory effects [18]. It is used as a drug delivery system in wound healing [19], cell encapsulation [20], implant preparation [21], gene carrying [22], and tissue engineering [23]. Furthermore, chitosan is sensitive to environmental stimuli [24]. In vaccine technology, its flexible structure and ability to be applied in different ways have made chitosan a potential antigen carrier [25,26,27]. In addition, chitosan-based products are also used for diagnostic purposes [28]. In this paper, the potential of chitosan-based nanogels in protein and gene applications is discussed comprehensively. The structural properties, preparation methods, modification strategies, and roles of chitosan nanogels in therapeutic areas, particularly in protein/peptide, antigen, and gene delivery, as well as in targeted carrier systems, have been evaluated in the light of current knowledge. In addition, the advantages of these systems, limitations, and future perspectives are discussed, together highlighting the role of chitosan nanogels as promising next-generation carrier systems in pharmaceutical technology and therapy.

## 2. Chitosan Nanogels

Nanogels have attracted considerable attention owing to their biocompatibility, gel-forming abilities, and structural properties. Numerous studies have concentrated on the synthesis of nanogels, which are 3D network structures at the nanoscale, using chitosan [29,30,31,32]. There are also reviews on nanogels [5,9,33] and chitosan nanogels [34,35,36,37]. The flexible structure, crosslinking ability, and gel-forming potential of chitosan has emerged as a highly attractive biopolymer in recent studies. The types of chitosan vary based on their molecular weight (MW) and degree of deacetylation (DD). In addition, the concentration, MW, and degree of acetylation of chitosan affect the characteristics of the gels formed. Various gel forms such as macroscopic hydrogel films [38], nanoparticle-based nanogels [39], and edible films [40] are prepared using chitosan.

## 3. Modifications on Chitosan

Chitosan’s limited solubility under physiological conditions, high viscosity, and batch-to-batch variability have prompted the development of various chemical and physical modifications. These modifications enhance its mucoadhesive, pH-sensitive, and nucleic acid-binding properties, which are critical for effective drug delivery applications. This section outlines key strategies to modify chitosan and enhance its performance as a nanogel-forming polymer.

Chitosan, which is obtained by removing some of the acetyl groups from chitin, dissolves in dilute acid solutions such as acetic and glutamic acid by protonating the –NH_2_ groups [41]. Chitosan, the only naturally occurring polycation, has a charge density influenced by pH and its degree of acetylation. While low MW oligomers dissolve over a broad pH range, high MW chitosan is only soluble in acidic conditions, even when highly deacetylated. This limits its use under neutral physiological conditions. To this end, several different types of chitosan derivatives have been produced [42].

The functional groups (hydroxyl and amine) on chitosan’s backbone enable it to form covalent bonds upon treatment with chemical crosslinkers [43]. By applying physical, chemical, and biological modifications to these chemical groups of chitosan, its solubility and stability can be changed. In this context, stimulus-sensitive systems have been developed with physical modifications using substances such as polyvinyl alcohol (PVA). In addition, structural changes were introduced using various chemical groups, such as chitosan derivatives (e.g., carboxymethyl chitosan), which form negatively charged and pH-sensitive gels. Thanks to these modifications, the efficiency of chitosan in drug transport systems increased significantly [44]. Furthermore, strong disulfide bonds in thiolated chitosan enhanced the polymer’s mucoadhesive properties [45]. Modified chitosan derivatives have been favorable drug carriers for negatively charged nucleic acids and proteins [46,47]. A new modification, which was developed by hybridizing positively charged chitosan with negatively charged proteins, yielded better drug loading and drug release characteristics compared to chitosan polysaccharide-based derivatives [48]. Additionally, chitosan derivatives can be mixed or crosslinked with organic components to develop modified chitosan nanogels [43]. A double enzyme-loaded multifunctional hybrid nanogel probe (superparamagnetic iron oxide@glycol chitosan/acrylic/biotin-catalase/superoxide dismutase-gel) was developed, enabling ultrasound imaging that responds to pathological stimuli and providing improved T2-weighted magnetic resonance imaging performance [49].

## 4. Chitosan Nanogel Production

A wide range of procedures, including physical gelation, ionotropic gelation, self-assembly, radical polymerization, and microemulsion, have been performed to develop chitosan nanogels depending on the desired pharmacological effect, properties, and dosage form, with each method differing in mechanisms and reaction conditions [43,50].

Chitosan, with a polysaccharide structure, is a unique polymer that is often used in the production of nanogels. Due to the presence of –OH and –NH_2_ groups, chitosan can engage in hydrogen bonding and electrostatic forces among its polymer chains, facilitating self-gelation and allowing nanogel formation without requiring surfactants, solvents, or crosslinkers [43,51]. The diameter of chitosan-based nanogels ranges from a few nanometers to 1000 nm. Factors including MW of chitosan, its DD, and the concentration of the polymeric solution significantly influence the polydispersity index and the thermosensitive properties of the nanogels. The covalent or ionic crosslinking, which occurs as a result of the formulation components and the production method, determines the structure of the nanogel [35,36].

Chitosan nanogels have the ability to transport drugs without being degraded or eliminated [52]. However, pure chitosan nanogels’ hydrophilic properties restrict their application in hydrophobic drug delivery [53]. The functionalization of chitosan with hydrophobic moieties, including alkyl, acyl, cholesterol, capric acid, cholic acid, deoxycholic acid, stearic acid, and 5β-cholanic acid, enables the encapsulation of lipophilic compounds within nanogel matrices [54,55,56,57,58]. Moreover, hydrophobic chitosan derivatives, such as carboxylated chitosan and chitosan sulfate, contribute to the amphiphilic character, enabling interactions with both anionic and cationic substances, promoting self-assembly, and enhancing water solubility, which in turn helps preserve nanogels against degradation and possible clearance [56].

The most crucial aspect of nanogels is their ability to be designed as stimuli-responsive drug delivery systems that react to internal and external factors, including temperature changes, ionic strength, light, pH, reductive reactions, and endogenous cellular enzymes [59]. Nanogels are capable of altering several physicochemical parameters, including their size, hydration level, refractive characteristics, porosity, and surface hydrophilicity, in response to environmental stimuli [60]. These inducing agents, whether environmental, endogenous, or externally applied, are used to direct nanocarriers to a specific target or activate it in a particular tissue, making stimuli-responsive nanogels critical in a wide range of conditions such as cancer [59,61].

At low pH, the protonation of –NH_2_ groups makes chitosan positively charged, enabling electrostatic interactions with negatively charged mucus or epithelial surfaces and thus providing mucoadhesive characteristics, rendering it well-suited for use in bioadhesive-based drug delivery systems [51]. Bioadhesive chitosan nanogels are mainly designed for oromucosal delivery (usnic acid) [62], periodontal delivery systems (flurbiprofen and triclosan) [63], dermal delivery (ketoprofen and madecassoside) [32,64], vaginal delivery (estradiol) [65], and ocular delivery (acetazolamide and resveratrol) [66,67]. Furthermore, by modifying these chitosan nanogels with pH-sensitive groups or particular ligands, therapeutic agents including genes, vaccines, and anticancer agents can be released to specific targets [36].

Depending on their bonding, nanogels are frequently crosslinked chemically or physically. Recently, nanogels incorporating both physical and chemical crosslinking approaches—referred to as hybrid nanogels—have gained considerable attention. While physical interactions often involve transient and weak bonds like hydrophobic associations, chemical crosslinking usually forms more stable and durable linkages, including covalent bonds [68].

### 4.1. Ionic Gelation

**Figure 3:**
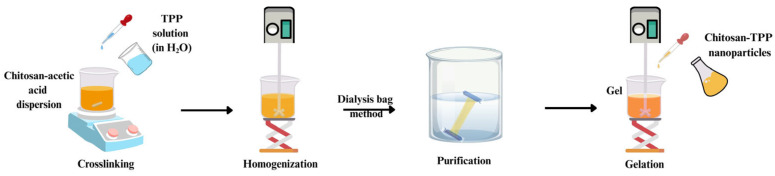
Ionic gelation technique for fabricating chitosan-based nanogels. This figure illustrates the preparation of chitosan-based nanogels using the ionic gelation method. The process begins with the addition of TPP solution into a chitosan–acetic acid dispersion under stirring, enabling ionic crosslinking. This is followed by homogenization and purification via the dialysis bag method. The resulting chitosan–TPP nanoparticles are subsequently incorporated into a hydrogel matrix, forming the final nanogel formulation.

Ionic gelation (Figure 3) is the predominant technique utilized in the fabrication of chitosan nanoparticles, in which polyelectrolytes interact with molecules of opposite charge, triggering a sol-to-gel transformation that facilitates the generation of delivery systems, including hydrogels and nanoparticles [69,70]. Tripolyphosphate (TPP) is by far the most frequently employed component for creating chitosan-based nanogels. This carrier system is obtained by ensuring ionic gelation of a gel network through ionic interaction between the cationic amine moieties of chitosan and the anionic phosphate moieties of TPP [71]. Experimental parameters, including MW and DD of chitosan, concentration of chitosan and TPP dispersions, mass ratio of chitosan to TPP, pH of the chitosan dispersion, reaction temperature, stirring speed, affect the fabrication of chitosan-TPP nanogels using the ionic gelation process [72]. Huang and Lapitsky highlighted that the particle size distributions of nanogels changed depending on TPP concentration, degree of chitosan deacetylation, and pH [73].

Ravi and Baskaran fabricated carotenoid fucoxanthin-loaded chitosan-TPP-glycolipid nanogels using the ionic gelation method, where nanoencapsulation of fucoxanthin improved its stability and bioavailability [74]. In another research, a chitosan-TPP-based nanogel containing folic acid, a compound with poor ocular bioavailability, was developed and reported as an effective carrier system. This nanogel enabled sustained and long-term drug release, helped maintain therapeutic folic acid levels, enhanced efficacy, and reduced dosing frequency [75]. Moreover, nanogels were developed by reacting chitosan with cashew gum for essential oil encapsulation [76], and with heparin via ionic gelation [77]. A unique three-component nanogel formulation composed of chitosan, ibuprofen, and gellan was engineered to function as a transdermal system, ensuring controlled release of ibuprofen by taking advantage of the chemical potential of drug-polymer electrostatic interactions. Ionic gelation and electrostatic nanoassembly methods were integrated to develop the ternary nanogels [78].

Besides TPP, glutaraldehyde is also frequently utilized as a cross-linking agent in hydrogel formation [79,80]. However, in recent years, enzyme-assisted gelling strategies have come to the fore due to safety concerns of toxic chemical crosslinkers [81,82]. To address this, Chen et al. [81] investigated enzyme-mediated gel formation. In their study, gelatin was used as the protein component and chitosan as the polysaccharide. Two enzymes, tyrosinase and transglutaminase, were tested for their abilities to catalyze in situ gel formation in protein-chitosan solutions. The researchers found that the gels prepared with tyrosinase exhibited limited mechanical strength. Moneira Teixeira et al. [83] explored intercalated hydrogels catalyzed by enzymes, including tyrosinase, transferase, and lysyl oxidase for tissue engineering applications. They reported that gels formed using tyrosinase were unstable and demonstrated weak mechanical properties. In another study, chitosan-glycolic acid/tyrosine (CH-GA/Tyr) conjugates can form gels quickly and safely by cross-linking via horseradish peroxidase or tyrosinase in biological environments. The CH-GA20/Tyr14 conjugate has shown high solubility up to pH 14. The gelation time was between seconds and minutes, depending on the enzyme and polymer concentrations. It has been reported that hydrogels crossed with horseradish peroxidase demonstrated higher storage modulus in rheological analyses, but gels crossed with tyrosinase had lower cytotoxicity in NIH/3T3 cells. Furthermore, it has been observed that cartilage cells in these systems had high viability rates for 14 days. These data show that enzyme-crosslinked chitosan gels are promising for multiple biomedical purposes, such as regenerative medicine and localized therapeutic delivery [82]. Lim et al. [84] developed binary (chitosan–gelatin) and ternary (chitosan–gelatin–nanohydroxyapatite) hydrogel systems using tyrosinase for scaffold fabrication and showed that hydrogels prepared with engineered tyrosinase were effective as injectable scaffold materials.

### 4.2. Physical Gelation

Physical gelation is a method applied to achieve gelation without using any cross-linking agent or organic solvent [85]. This method relies on reverse emulsion of a chitosan dispersion in a typically safe surfactant system [86,87]. The physical gelation of chitosan occurs via weak intermolecular forces, including hydrogen bonding, charge-based interactions, and hydrophobic effects. These interactions respond to changes in environmental conditions such as pH, temperature, and ionic strength, as well as the addition of valuable counter ions. Chitosan can interact with oppositely charged ions and molecules by acting as a polyelectrolyte in pH 4–6. Compared to chemical crosslinked gels, physical chitosan gels are much safer since they do not contain toxic crosslinkers, and their decomposition properties can be adjusted by swelling. However, their mechanical strength is usually lower. In addition, the DD of chitosan and the cosolvents found in the system have significant effects on gel formation and stability. Recent studies have shown that gelation kinetics might be adjusted by precise control of formulation parameters, which makes physical gels attractive for drug delivery systems [88]. While the current knowledge about physical gelation strategies at the nanoscale has remained limited, ongoing research continues to reveal their potential in developing biocompatible and environmentally friendly nanogel platforms for controlled drug delivery [43].

### 4.3. Self-Assembly

The self-assembly method is a convenient approach for fabricating nanogels, allowing the establishment of hydrogen, ionic, and covalent bonds, alongside electrostatic and van der Waals forces [89,90]. Self-assembly is a simple and versatile technique compared to other methods. To create chitosan-based nanogels through self-assembly, many organic and inorganic components including peptides, proteins, alcohols, organic acids, and salts have been used. Acids are commonly employed to react with chitosan, leading to the formation of amide bonds between the -COOH moieties of the acids and the -NH_2_ moieties of chitosan [43]. Such acids comprise ethylenediaminetetraacetic acid [91], benzoic acid [92], caffeic acid [93], cinnamic acid [94], azelaic acid [41], folic acid [95], and maleic acid [96]. Self-assembled chitosan–acid-based nanogels demonstrated promising prospects for biomedical applications and exhibited favorable circulation half-life along and efficient systemic clearance [43]. Peptides (e.g., exendin-4 and glutathione) and proteins (e.g., ovalbumin and bovine serum albumin) may also undergo self-assembly with chitosan via electrostatic interactions to produce nanogels [14,57,97,98].

Nanogels formed by self-assembly of native polysaccharides offer an effective drug delivery platform for various therapeutic compounds, maintaining the physicochemical and biological characteristics of the polysaccharides without requiring chemical functionalization. Using this method, many anionic drugs, including small molecules, nucleic acids, and proteins, have been encapsulated effectively [13]. Sodium chloride is added during gel formation to help restrict the nanogel size distribution and increase stability [73,99]. The drug should be delivered precisely to the site of action. Multiple advantages are offered by self-assembled polysaccharide-based nanogels to help overcome some of the biological barriers [13].

### 4.4. Radical Polymerization

Radical polymerization is a process in which polymers are synthesized through the successive addition of radicals to monomer units, with these reactive radicals commonly generated by different initiation mechanisms involving specific initiator compounds [100]. In this approach, synthetic polymers are grafted onto natural polysaccharides like chitosan or chitin to produce interpenetrating polymeric networks through free radical polymerization of vinyl- and acrylic-based monomer units. Chitosan, serving as a polymeric ionic template, is commonly employed with ionizable monomers including acrylic and methacrylic acids, acrylamide, and their structural variants for macromolecular template synthesis [101,102,103].

Recent advancements in precision polymer synthesis enabled by controlled radical polymerization techniques have opened up significant potential for tailoring polymeric architectures with high flexibility, multifunctionality, and structural complexity. Because of their adaptability, these systems might self-assemble into a variety of nanoscale objects with different sizes and shapes [104]. This approach is employed to develop nanogels in the presence of flexible crosslinkers [5]. After polymerization, the polymer chains begin to elongate until they reach a critical insoluble length, eventually resulting in the formation of stable, dispersed polymeric nanogels [105]. This approach is well-suited for generating nanogels with varied compositions, dimensional profiles, and structural characteristics.

Radical polymerization is a powerful strategy to produce chitosan-derived nanogels [106]. Researchers have recently developed thermoresponsive and smart pH-sensitive nanogels via radical polymerization. Poly(N-isopropylacrylamide) (PNIPAAm), a thermoresponsive smart polymer with a lower critical solution temperature (LCST) of 32 °C, can be copolymerized in combination with chitosan or its derivatives, enabling the formation of nanogels responsive to pH or temperature. In one study, copolymers of chitosan grafted with poly(N-isopropylacrylamide) (CS-g-PNIPAAm) exhibiting thermoresponsive behavior were prepared in aqueous medium through a free radical polymerization process initiated by potassium persulfate. By adjusting the molar ratio of CS to NIPAAm within the range of 1:0.25 to 1:10, copolymers with varying PNIPAAm chain densities were synthesized. Aqueous solutions of copolymers containing more than 12 mol% of PNIPAAm demonstrated thermoresponsive characteristics, undergoing separation into distinct phases at approximately 29.0–32.7 °C. The thermal sensitivity increased as the PNIPAAm content in the copolymer increased. These copolymers hold great promise for use in drug delivery systems and tissue engineering strategies [107].

In addition to responding to pH and temperature changes, these nanogels might markedly enhance the effectiveness of loaded drugs compared to free drugs [108]. However, numerous organic and inorganic materials can be generated throughout the radical polymerization process, potentially leading to unexpected byproducts that may compromise the integrity or performance of the resulting nanogels [43].

### 4.5. Microemulsion Technique

**Figure 4:**
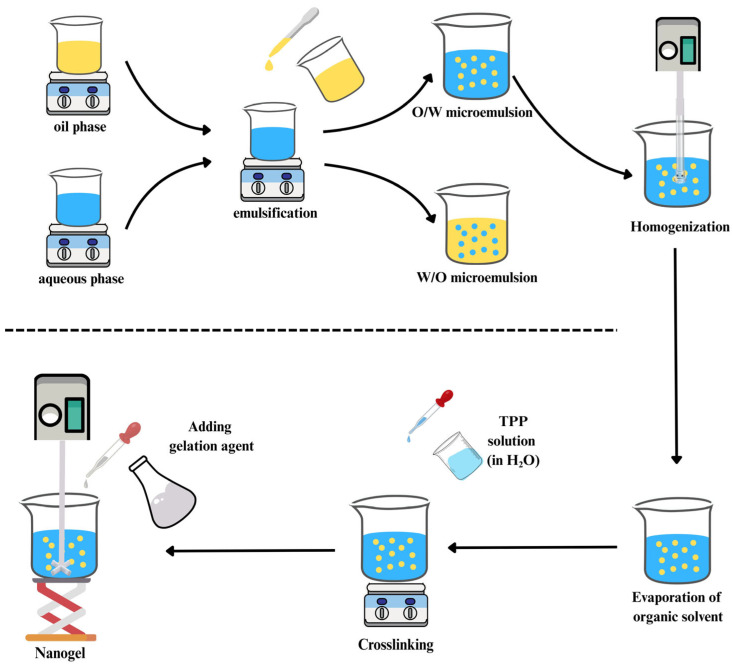
Microemulsion technique for fabrication of chitosan-based nanogel.

Microemulsion enables two immiscible phases to form nano-sized droplets with the help of surfactants and is thermodynamically stable [109]. This method involves adding crosslinkers and chitosan solution to an organic solvent containing surfactants. To complete the crosslinking, the resulting mixture is agitated overnight. Afterwards, air drying or freeze-drying is used to remove the organic solvent (Figure 4). The main disadvantages of this method are its lengthy process, potential toxicity, and the use of organic solvents [110].

An atorvastatin-black seed oil-loaded chitosan-carboxymethyl nanogel was developed using the microemulsion technique. Chitosan was dispersed in an acetic acid medium and blended with an aqueous solution of PVA. Meanwhile, the substances forming the organic phase (atorvastatin, Nigella sativa oil, glycerin, and Span 80) were dissolved in ethanol. A microemulsion was formed by homogenizing the two phases. After the organic solvent was evaporated, crosslinking was enabled by adding TPP solution to this mixture, followed by adding and mixing carboxymethyl chitosan (CMC) [111].

To improve the ocular availability of loteprednol and sustain its therapeutic concentration locally over a prolonged duration, a novel biodegradable and mucoadhesive polymer nanoparticle-based nanogel system was developed. Carbodiimide chemistry was used to graft the oil/water-produced poly(butylene adipate) nanoparticles onto N-succinyl chitosan. Concurrently, the chitosan layer, serving as the shell component, self-crosslinked to stabilize the poly(butylene adipate) chitosan nanoparticles. The findings of release studies indicated that the nanogels may be highly useful for delivering hydrophobic drugs for improved ocular treatment [112].

In another study, it was reported the fabrication of chitosan-based hydrogels at both macroscopic and nanoscopic scales through gamma radiation-induced grafting of N-vinylcaprolactam. The crosslinked chitosan nanogels were obtained using the reverse microemulsion technique (water/oil). The effectiveness of these delivery systems was evaluated using 5-fluorouracil as the model drug. Both macro and nanogels responded to temperature (31–33 °C) and pH (4.75). However, nanogels responded to both stimuli with greater swelling due to their larger surface areas. Both systems demonstrated that the modified macro- and nanogels might be viable alternatives as drug delivery platforms, maintaining release for over 12 h [113]. Although microemulsion is a promising approach for the routine preparation of nanogels, the necessity of large amounts of surfactants and solvents limits its industrial-scale production [43]. Additionally, synthetic surfactants such as Triton X-100, which are widely used in conventional microemulsion systems, are critical for environmental toxicity and biodegradability. Therefore, in recent years, biosurfactants obtained from microorganisms such as *Bacillus subtilis* (for example, lipopeptides and glycolipids) have come to the fore as sustainable and biocompatible alternatives for green nanotechnology applications. These compounds offer low toxicity, environmental safety and high surface activity in pharmaceutical formulations. The integration of such biosurfactants in microemulsion-based nanogel production processes has the potential to increase patient safety and reduce the environmental burden in industrial production [114].

## 5. Smart Stimuli-Responsive Chitosan Nanogels

**Figure 5:**
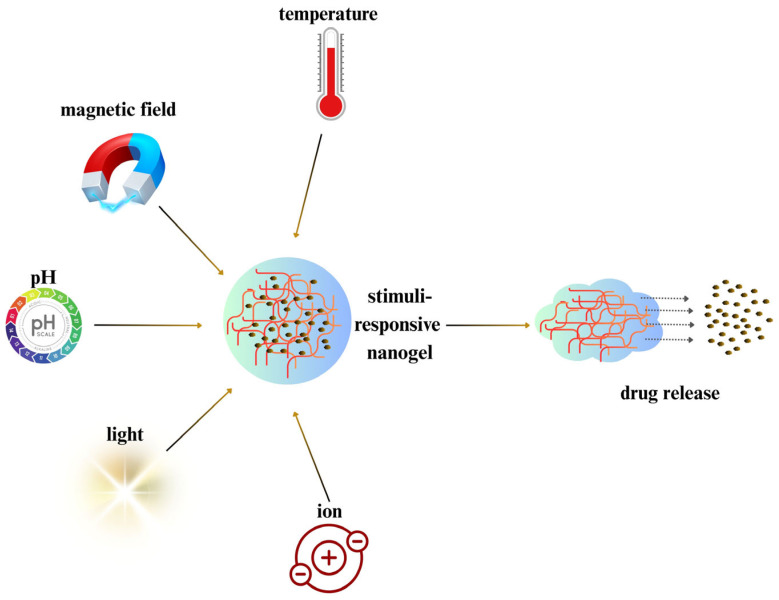
Environmental triggers activating cargo delivery via stimuli-responsive nanogels. Stimuli-responsive nanogels can swell, degrade, or alter their conformation in response to these triggers, providing spatiotemporal control over drug delivery.

Stimulus-sensitive nanogels change their physicochemical characteristics such as water content, volume, refractive index, and hydrophilicity/lipophilicity, triggered by alterations in environmental factors, including pH, light, temperature, etc. (Figure 5). Their abilities to encapsulate bioactive molecules, remain highly stable during long-term circulation in the bloodstream, target specific sites, and provide controlled drug release modulated by environmental conditions make stimuli-responsive nanogels unique among polymeric nanoparticles used in drug delivery.

This approach offers promising opportunities, especially as nanogels have evolved from passive carriers into active participants in disease treatment optimization [60]. Controlled drug release is allowed by the nanogels’ responsiveness to internal or external conditions such as temperature, pH, light, and redox conditions. This “smart” targeting capability reduces adverse effects and avoids accumulation in non-targeted organs [59]. In this context, the functional groups in chitosan’s structure provide a practical and reliable advantage [115]. It has been demonstrated that pathological conditions such as lesions or injuries cause alterations in pH, temperature, and enzyme levels compared to healthy tissues. These variations allow the design of effective, tissue-specific delivery systems, minimizing adverse effects and enhancing therapeutic efficacy in regenerative medicine. The development of on-demand delivery platforms involves using chemical triggers or external stimuli to regulate the timing and intensity of active ingredient release. The characteristics of various chitosan nanogels in response to different stimuli have yielded highly successful results in drug delivery [116].

One study demonstrated that a green chemistry method could conjugate a hydrophobic drug molecule (Lilial) with biocompatible and biodegradable chitosan. This modification not only improved chitosan’s modifiability and solubility but also made it sensitive to stimuli-responsive cleavage. Thermosensitive poly(N-isopropylacrylamide) was grafted onto the chitosan–Lilial conjugate. As a result, the nanocarriers disintegrated and disassembled into reversed micelles exhibiting significantly reduced dimensions (~8 ± 3 nm), favoring renal clearance under biomimetic conditions resembling endosomal or lysosomal trafficking routes (T = 37 °C, pH = 4.5). Within 30 h, 70% of Lilial molecules were released via hydrolytic cleavage of the exposed Schiff base bond. This stimuli-triggered release behavior revealed an effective method to prepare chitosan-based nanocarrier systems for intravenous delivery of poorly water-soluble drugs [117].

In another study, an intestinal enzyme-sensitive nanogel was developed for long-term controlled release of imatinib, a hydrophobic anticancer agent. Methacrylic anhydride-modified carboxymethyl chitosan (MA-CMCs) was fabricated by amidating carboxymethyl chitosan with methacrylic anhydride, followed by UV-induced crosslinking in the presence of a photoinitiator to yield a 3D hydrophilic network. Enzyme-triggered hydrolysis of glucosidic bonds enabled imatinib release, while sodium deoxycholate embedded in the hydrogel helped to open epithelial tight junctions and to improve intestinal permeability. Oral administration of imatinib-loaded hydrogels led to a significant enhancement in tumor inhibition, achieving a six-fold increase compared to imatinib mesylate [118].

In another example, chitosan nanoparticles designed for the co-delivery of 5-fluorouracil and polypyrrole, and coated with CMC, were developed to enable a synergistic effect of chemotherapy and photothermal therapy. The produced nanoparticles had an average size of 254.4 nm and a drug content of 24.2% and displayed a spherical shape. Controlled drug release was achieved by multi-modal NIR irradiation, triggered by the tumor’s high glutathione levels and acidic microenvironment. The nanoparticles were effectively internalized by HepG2 cells and demonstrated strong tumor suppression in tumor-bearing mice [119].

## 6. Chitosan Protein Hybrids

The most recent and cutting-edge nanomaterials are hybrids, or nanocomposites, composed of both polymeric and inorganic components. The goal of creating these nanocomposites is to combine the best qualities of both constituents and enhance their functional activities [120]. Proteins and polysaccharides, as examples of natural biomacromolecules, are generally preferred to synthetic alternatives due to their biocompatibility, biodegradability, sustainability, and environmentally friendly features [121]. Protein–polysaccharide composites, which can be produced in various forms such as patches, fibers, particles, and gels, have broad applications in both biomedical sciences and green chemistry as new biocompatible and biodegradable polymeric materials [122]. Polymer–protein hybrid systems have significant characteristics including non-toxicity, non-irritancy, and high water solubility [123]. They offer several advantages including increased solubility and stability, superior emulsion properties, extended circulation time, diminished immunogenicity, and enhanced specificity in directing delivery to target tissues compared to unmodified peptides and proteins [124,125].

Chitosan, a promising natural polysaccharide, might be used in protein-polysaccharide composites for hybrid nanogel formulations [126]. The combination of its physical, chemical, and biological features enables its suitability for assembling proteins, nucleic acids, and viral particles [127]. The primary amine located at the C2 position of glucosamine units provides chitosan with a distinct structural characteristic and enables its conjugation with proteins and peptides. When conjugated with proteins, chitosan exhibits unique properties such as swelling, self-assembly, and pH responsiveness [126].

Hybrid nanogels intended as targeted carriers may serve in applications including cell imaging, tissue engineering, insulin delivery, and antidiabetic dietary research. Utilizing a combination of freeze-drying, microreplication, and rapid prototyping, researchers created a chitosan–gelatin scaffold characterized by its uniform architecture and high porosity for hepatic tissue engineering. Histological and SEM analyses showed that hepatocytes grew into sizable colonies within the designated hepatic chambers, filling the cavities completely over a seven-day culture period [128].

In a study, papain was used to develop a protein-carrier hybrid nanogel system. It was encapsulated in chitosan-based and chitosan–PEG composite nanohydrogels. Various techniques were employed to evaluate gel properties. The combination of 0.75% chitosan and 1% sodium TPP resulted in the highest level of papain encapsulation. Additionally, the incorporation of polyethylene glycol reduced the melting point of the hybrid nanohydrogels, contributing to the development of more flexible structures. The highest papain release was observed at elevated temperatures and pH values below 7. Moreover, polyethylene glycol enhanced the sustained release of papain under physiological-like conditions, especially at lower PEG ratios. These findings suggest that the structure and release profile of hybrid nanohydrogels make them promising candidates for protein delivery systems [129].

Achieving effective drug concentrations at the target site remains a significant challenge for drug delivery in full-thickness skin wounds. In a related study, a protein-polysaccharide-based composite hydrogel was developed to provide controlled drug release. Nanogels containing spirulina protein, known for its antioxidant, anti-inflammatory, and signaling modulation properties, were integrated into a carboxymethyl chitosan-based hydrogel network. This system demonstrated rapid gelling, high mechanical strength, and adjustable drug release upon variation in the ratio of formulation. The nanogel demonstrated strong antibacterial effects against both Gram-positive bacteria (99.77% *Staphylococcus aureus*) and Gram-negative bacteria (98.64% *Escherichia coli*), along with good biocompatibility. It also reduced inflammation and oxidative stress. Wound healing accelerated, with healing rates of 78.37% on day 7 and 99.13% on day 12, relative to 56.99% and 84.50% in the control group. This effect was associated with reduced inflammation, granulation tissue formation, collagen accumulation, and enhanced angiogenesis. Thus, this protein-polysaccharide-based composite nanogel offers a promising option for wound healing and drug delivery [130].

Chitosan and ovalbumin, both natural macromolecules, are frequently utilized to fabricate nanogels by a novel, green, and practical technique. Chitosan and ovalbumin solutions were blended, the pH was adjusted, and the mixture was stirred and heated. Upon heating, ovalbumin formed nanospheres through gelation. These nanogels maintained their size distribution during long-term storage and remained stable between pH 2 and 10.5. Their distribution capacity, sizes, and hydrophilic/hydrophobic balances were pH-dependent. These nanogels are promising targets in cosmetic and pharmaceutical applications [98].

Chondroitin sulfate is a naturally occurring, negatively charged macromolecule that belongs to the glycosaminoglycan family and is widely found in the extracellular matrix. It is important to maintain the structural integrity of tissues. Owing to its anti-inflammatory, antioxidant, and anti-apoptotic properties, it has been extensively employed in promoting bone health and treating dry eye syndrome and is also highly suitable for chitosan-based nanogel formulations. In one study, a nanogel formulation incorporating low MW chitosan and chondroitin sulfate was prepared using the naringenin–β-cyclodextrin (NAR/β-CD) complex to address early-stage diabetic retinopathy. Among the tested candidates, the N3 system, characterized by favorable biocompatibility, nanoscale dimensions (<350 nm), and a positive surface charge, was chosen for NAR/β-CD encapsulation in the final N3@NAR/β-CD construct. This formulation enabled sustained drug release over 72 h. The physicochemical properties of N3@NAR/β-CD facilitated retinal endothelial cell uptake and have shown promise in reducing injection frequency while maintaining therapeutic levels in the damaged retinal region [131].

One way to improve gene transfer efficiency with chitosan-based nanoparticles is to add guiding caryophilic peptides to the nucleus in the transporters. In a study, the authors examined how variations in the length and sequence of caryophilic peptides (P1 and P2) influence the physicochemical and biological behaviors of plasmid-loaded nanoparticles. In chitosan-based nanoparticles prepared with pEGFP-N1, pQBI25, and pSelect-Zeo-HSV1-tk plasmids, the particle size decreased when P2 peptide was used, whereas the nucleus uptake and green fluorescent protein expression increased significantly. There was no significant change in polydispersity and zeta potential. As a result, the sequence and length of caryophilic peptides were shown to affect the performance of chitosan-based transporters used for gene transfer [132].

## 7. Protein–Peptide Delivery

Proteins are traditionally administered parenterally due to their structural instability, susceptibility to enzymatic degradation, and poor absorption. To overcome these challenges, protein and peptide carriers, including nanogels, have been developed to protect them from harsh environments such as enzymes and acidic conditions.

The release of peptides and proteins from nanogels may occur through diffusion or be triggered by internal or external stimuli [133,134]. Drug release might be regulated by adjusting the density of the polymer chains [135]. Moreover, one of the most important strategies has been the release of proteins through nanogel degradation and network dispersion [136]. The intensity of crosslinking also affected drug release by influencing the nanogel size [137]. Unlike liposomes or insoluble nanoparticles, hydrophilic nanogels swell during drug release. This swelling allows sustained drug release from the inner layers as the outer layers deplete. As a result, continuous protein release at the disease site might be obtained [133]. Additionally, nanogels might encapsulate higher amounts of drugs compared to other nanoparticles and support sustained release of these molecules [135].

Triggers to enable release from protein-loaded nanogels include pH changes, external energy input, or charge interactions with counterions [133]. For instance, a nanocomplex gel synthesized from glycol chitosan and 2,3-dimethylmaleic anhydride (DMA) formed a core–shell complex with lysozyme at pH 7.4. At sub-physiological pH values indicative of mild acidity (pH 6.8), charge reversal due to DMA separation increased electrostatic repulsion, triggering lysozyme release [138].

Hollow nanogels made from glycol chitosan and aldehyde-capped PEG via a one-step ultrasonic spray method were used to encapsulate urokinase-type plasminogen activator (uPA). These nanogels (200–300 nm) extended circulation time and released uPA more rapidly under diagnostic ultrasound (2 MHz), significantly enhancing clot thrombolysis [139].

A novel approach developed for the treatment of osteoarthritis focused on using functional nanogels that provide long-term drug release into the joint. In a study, nanogels based on chitosan and hyaluronic acid, functionalized with endothelin and bradykinin receptor antagonists, were developed. In the OA model created with IL-1β, the combination of these nanogels reduced inflammation and tissue destruction in the most effective way. These findings showed that this system has shown promise in the treatment of osteoarthritis [140].

A major contributing factor to complications in orthopedic implants is infection, and antibiotic-resistant bacteria play a critical role in this process. Costa et al. developed antimicrobial nanogels (Dhvar5-NG) to be applied on-site to the implant surface. Dhvar5 (LLLFLLKKRKKRKY), a broad-spectrum antimicrobial peptide, has been bound to chitosan by “thiol–norbornene photoclick” chemistry and converted into nanogel form by a microfluidic system. Cytotoxicity was not observed in tests with pre-osteoblast cells. In addition, the combined effect of Dhvar5-NG with antibiotics (piperacillin and oxacillin) has been studied. At a concentration of 10^9^ nanogels/mL, Dhvar5-NG provided a 4-fold (2 → 0.5 µg/mL) increase in the effect of oxacillin and a 2-fold (2 → 1 µg/mL) increase in the effect of piperacillin. These results suggested that Dhvar5-NG may help antibiotics in reducing the risk of infections associated with orthopedic implants [141].

In another study, polycaprolactone (PCL) fibrous mats underwent distinct surface modifications to facilitate the incorporation and controlled release of human bone morphogenetic protein 2 (BMP-2), followed by chitosan nanogel functionalization. Three different surface modifications were evaluated in terms of BMP-2 binding capacities and release profiles. Polydopamine coating was the system that bound the most BMP-2 according to surface area, but protein release was found to be limited. In contrast, BMP-2 binding was reduced on PCL mats that had been coated with chitosan-grafted PCL and further layered with alginate, followed by surface modification via air plasma, but was able to release almost all of it. The incorporation of BMP-2 within the chitosan-based nanogel formulation improved the adherence to various surfaces and ensured a regulated delivery, maintaining its biological function [142].

The molecular chaperone-like effect of myristic acid and chitosan-based nanogels (CMAs) has been studied by a refolding model of the neuroserpine protein from the serine proteinase inhibitor family. By creating amide bonds via the 1-ethyl-3-(3-dimethylaminopropyl) carbodiimide-mediated process, myristic acid and chitosan covalently bound together. In tests conducted at different temperatures (25–80 °C), the structural distortions of neuroserpine significantly reduced in the presence of CMAs. The CMAs prevented heat-induced aggregation of the protein and exhibited chaperone-like behavior. CMAs are effective in maintaining protein structure and have therapeutic potential in neurodegenerative disorders such as Alzheimer’s, Parkinson’s, Huntington’s diseases, and prionopathies [143].

Hybrid nanogels, which are cross-linked physical and chemical composite gels, were developed by Wu and associates to co-deliver doxorubicin, the injectable recombinant cytokines interleukin-2 and interferon-gamma [144].

Nisin’s broad antibiotic effectiveness against Gram-positive bacteria and its low toxicity profile made it quite interesting. In order to increase its prolonged antibacterial effect, nisin-loaded nanogels have been developed with carboxymethyl chitosan. These nanogels were obtained by electrostatic self-assembly and chemical cross-linking methods. They had spherical structures and were well-distributed. The average particle size was around 45 ± 5.62 nm. Hydrogen bonds and ionic interactions between nisin and carboxymethyl chitosan formed the primary factor promoting nanogel formation. The nanogel controlled nisin release and showed strong antimicrobial activity against foodborne bacteria. These findings indicated that this nanogel system might be employed as a potential natural preservative candidate in the food industry [145].

To improve the ability of chitosan, thiol moieties could be grafted onto the chitosan. Thus, thiolated chitosan derivatives were developed [45]. Guaresti and colleagues synthesized a nanogel system through a reverse emulsion technique based on cyclohexane/Triton X-100/n-hexane/water mixtures through covalent bonding between chitosan’s primary amine groups and a dicarboxylated poly(ethylene glycol) cross-linker, then labeled it with folate ligand. Chitosan-labeled nanogels demonstrated a tunable behavior to the environmental factors due to their dual pH responses and mucoadhesive capacities, which altogether made them successful candidates for biomedical applications [146].

Zhang et al. developed a composite nanogel system (CMC@CMCS@OPs) by incorporating oyster peptides (OPs) into a matrix composed of carboxymethyl chitosan (CMCS) and CMC. Exhibiting a compact spherical morphology with an average diameter of approximately 64 nm, the nanogel enhanced the thermal and enzymatic stability of OPs and effectively preserved their hypoglycemic activity. Moreover, the system’s swelling behavior and drug release profile confirmed its potential for targeted and sustained intestinal delivery. It was not toxic to Caco-2 cells. It had an increased permeability coefficient of 4.70–7.45 times and an improved absorption rate of 129.38% compared to OPs. The transition of the nanogel from the cell (transcytosis) was mainly carried out by macropinocytosis, endocytosis, and intestinal carrier-mediated efflux pathways. As a result, the CMC@CMCS@OPs nanogel is promising as an effective carrier system that increases the stability and bioavailability of OPs [147].

Chitosan nanogels have long been appreciated in insulin delivery. In a study, two different insulin-loaded nanogel formulations with opposite surface charges (−15.94 mV for insulin: carboxymethyl chitosan (CMCS)/chitosan (CS)−nanogels(−), +17.15 mV for insulin: CMCS/CS-NGs(+)) were fabricated within the scope of the insulin transporter system developed for the treatment of diabetes. In ex vivo studies, it was determined that nanogels with opposite surface charges showed distinct adhesion and transition properties in intestinal segments. While no statistically significant differences were noted in the rat duodenum, insulin: CMCS/CS-NGs(−) showed approximately 3 times more adhesion and 1.7 times more transition in the jejunum than insulin: CMCS/CS-NGs(+). These findings revealed that the surface charges of the nanogels determine the absorption zones in the small intestine. In an in vivo study, blood sugar levels were determined to be 3 mmol/L lower in the insulin: CMCS/CS-NGs(−) group between one hour and 11 h after oral application compared to the insulin: CMCS/CS-NGs(+) group. Moreover, the insulin: CMCS/CS-NGs(−) group showed that negatively charged nanogels were more effective in controlling blood sugar [148].

In another study, chitosan-based luminescent/magnetic (CLM) hybrid nanogels were fabricated using chitosan, quantum dots for cell imaging, and superparamagnetic iron oxide. Insulin loading was performed using spherical CLM nanogels with suitable average diameters (<160 nm). About 40.1 mg/g of insulin was the actual loading dose. Insulin-loaded CLM nanogels were employed to examine how compounds like mangiferin, (−)-epigallocatechin gallate, and (−)-epicatechin gallate influence cellular uptake through insulin receptor pathways in normal human hepatocyte L02 cells [149].

Compounds derived from seafood have been intensively investigated to be used in therapeutic approaches owing to their biological activities. Due to its superior bioadhesion ability and solubility properties, chitosan is a biopolymeric delivery platform widely used in the transportation of peptide, protein, and similar nutritional supplements. In a study, modified thiol-based chitosan-based nanoparticles were developed to transport an anti-inflammatory peptide (ESPVL) obtained from sea crab. These nanoparticles provided controlled release for 30 h, showed high thermal stability and did not produce toxic effects up to 100 µM. In addition, lipopolysaccharide-stimulated cells decreased the generation of nitrite and reactive oxygen species, suppressed inflammation-related cytokines and p-p65 levels. Molecular interaction analyses have shown that this system had high stability and anti-inflammatory effect [150].

## 8. Antigen Delivery

Chitosan has been used as an effective carrier for vaccine adjuvants and nano-vaccine delivery systems due to its good biodegradability, biocompatibility, low toxicity, immune-stimulating activity, targeting localization, and ability to provide gradual or controlled release of vaccines. In addition to improving drug penetration and regulating release, chitosan-based nanogels also serve as antigen reservoirs, immunological modulators, and antigen-presenting platforms. For vaccine delivery, chitosan-based hydrogels are particularly helpful and feasible due to their water solubility and pH sensitivity. Owing to these properties, pH-sensitive chitosan nanogels with low immunogenicity and high biosafety are considered potential candidates for vaccine delivery systems [35].

In one study, phosphorylated chitosan (PCS), a water-soluble chitosan derivative, was synthesized and assessed for use as an innovative immune adjuvant. PCS demonstrated pH sensitivity: it remained in solution at pH < 7.0 and transitioned into a gel state at pH > 7.0. Owing to this behavior, a neutral PCS solution containing the ovalbumin antigen formed a gel matrix upon intramuscular injection into mice, enabling prolonged antigen retention at the injection site and stimulating a strong immune response. Using 30 mg/mL PCS-based hydrogel significantly increased the levels of CD4^+^ and CD8^+^ memory T cells, along with secreting antigen-specific IgG antibodies, IFN-γ, and IL-4 cytokines from splenocytes. In vivo imaging and immunohistochemical analyses indicated that the increased immune activity was likely due to the controlled release of the antigen and its prolonged interaction with immune cells via the PCS gel matrix. These findings suggested that PCS is a promising vaccine carrier system in immunotherapy [151].

Neospora caninum (Nc) is an obligate intracellular parasite. In a study examining the protective effects of nanogel-encapsulated recombinant NcPDI (recNcPDI), mice were inoculated intranasally or intraperitoneally and later challenged with Nc tachyzoites. The chitosan-based nanogels featured alginate or alginate–mannose surface coatings. Intranasal administration of recNcPDI-loaded nanogels protected 9 out of 10 mice, whereas none of the animals that received intraperitoneal injections without nanogels survived. Interestingly, nanogels without recNcPDI offered some level of protection, but the combination of nanogels and recNcPDI notably improved survival following intraperitoneal challenge. Overall, 80% of the mice were protected after receiving the recNcPDI-loaded nanogels [152].

## 9. Gene Delivery

Gene therapy is a technique to transfer foreign genetic material into host cells to correct existing dysfunctions or support relevant cells with new functions to achieve a therapeutic benefit. While gene therapy initially addressed rare genetic disorders, the idea of delivering nucleic acids, such as messenger RNAs (mRNAs), plasmid DNAs, and small interfering RNAs (siRNAs), to silence or restore a particular gene function has been applied in the treatment of various diseases. Nucleic acids, however, are quickly eliminated from the bloodstream, exhibit enzymatic and chemical instability, and often face challenges in crossing cellular membranes. Furthermore, the necessity of an appropriate carrier system has been the most challenging step in resolving these issues and choosing the best course for efficient gene delivery. Chitosan-based nanogels for gene delivery are typically prepared using gentle aqueous-based techniques, such as ionic gelation or polyelectrolyte complexation, which avoid harsh conditions like high temperatures, organic solvents, or sonication. These mild formulation methods are particularly suitable for loading sensitive genetic materials such as siRNA, mRNA, or plasmid DNA, as they preserve the structural integrity and biological functionality of the nucleic acids. Several studies have confirmed that these systems can encapsulate and protect oligonucleotides without causing significant degradation during the preparation process, supporting their suitability for non-viral gene delivery applications [153,154,155,156].

**Figure 6:**
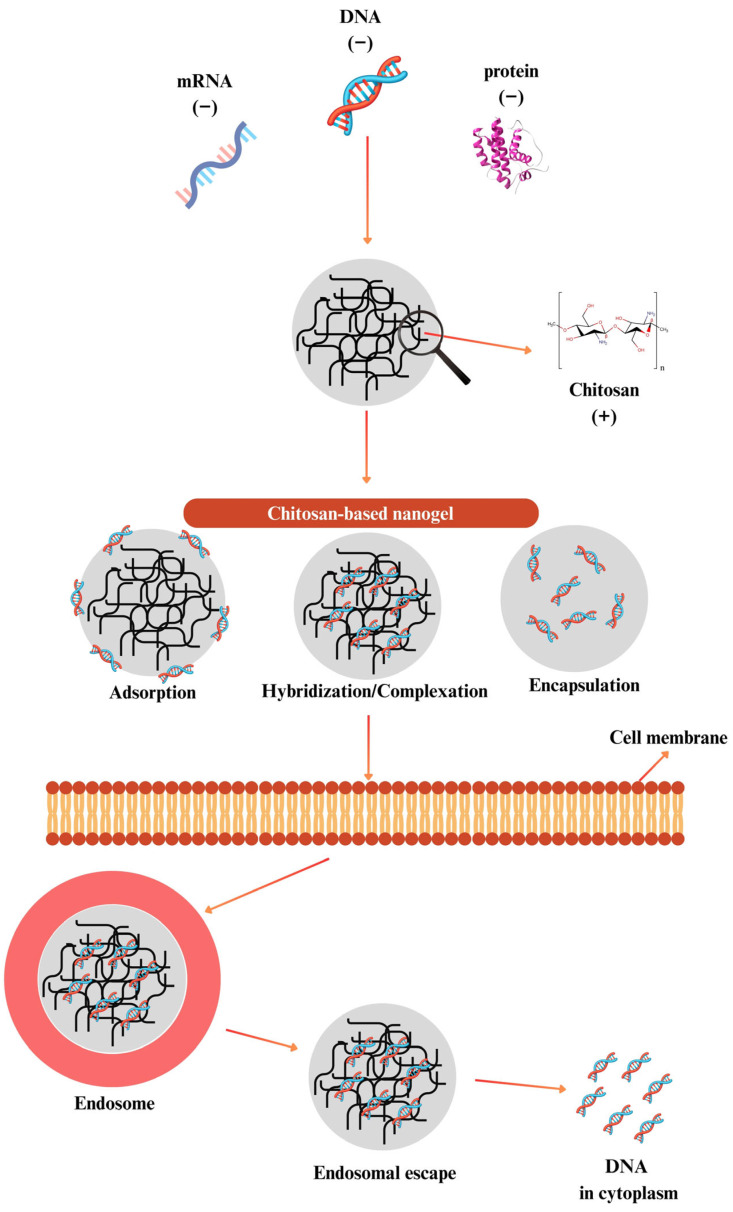
Chitosan nanogels for gene and protein delivery: Loading strategies and intracellular transport. Chitosan-based nanogels, due to their cationic nature, effectively interact with negatively charged biomacromolecules such as DNA, mRNA, and proteins through various mechanisms, including adsorption, complexation/hybridization, and encapsulation. These nanogels are internalized by cells via endocytosis, and once inside, they accumulate in endosomes. Due to the proton sponge effect of chitosan, the nanogels might escape the endosomal compartment, releasing their cargo into the cytoplasm. This figure illustrates the sequential process of loading, cellular uptake, endosomal escape, and final delivery of biomolecules using chitosan-based nanogels.

Chitosan-based formulations might provide this escape, which is attributed to the “proton sponge effect”. In this mechanism, chitosan, with pKa value of 6.5, gains a positive charge by being protonated more and more in the endosomal acidic environment. These increased cationic loads cause water and chloride ions to enter the endosome, which leads to osmotic swelling and, as a result, physical rupture of the endosomes. Thus, chitosan-based carriers might release into the cytoplasm and deliver the product they carry to the target without any degradation [157,158] (Figure 6). This capability has enabled the use of chitosan as a gene delivery system for delivering genetic materials such as DNA [153], RNA [159], oligonucleotide [160], siRNA [161], miRNA [162], and aptamer [163] for many years [164,165]. Moreover, chitosan has the ability to easily form complexes with nucleic acids. However, it is important to assess the characteristics that gene delivery systems should have before discussing how well nanogels work as a gene delivery method.

An ideal gene delivery system is expected to effectively encapsulate nucleic acids while protecting them degrading agents such as nucleases [166]. Nanogels might encapsulate high amounts of hydrophilic and hydrophobic cargos [167]. Furthermore, because of its cationic characteristic, chitosan binds oppositely charged nucleic acids and makes it easier for them to enter the cell by allowing passage through the negatively charged cell membrane. Another feature sought in a gene carrier is to carry nucleic acids to the target area. Nanogels can deliver molecules to the target cell via passive targeting and target-appropriate ligand connections [168]; thus, the drug effect is provided in the diseased tissue while the healthy tissue is not affected.

The persistent variability of the formulations used is a drawback of nanogels. Even though the improvements in nanoscale manufacturing made it possible to control the distribution of particle sizes more precisely, it is still challenging to precisely reproduce the size and stoichiometry of nanogels [9]. Chemical grafting onto a polysaccharide creates self-assembled glycol chitosan nanogel. The addition of a folate moiety to the nanogel enhanced its interaction with the folate receptor and encouraged endocytosis for internalization. This carrier system complexed siRNA efficiently [169].

In a study, a new nanolipogel (NLG) system, created by the encapsulation of siRNA/chitosan-methacrylate complexes into liposomes and capable of cross-linking with UV, was evaluated. It has been shown that this system was able to encapsulate siRNA effectively and provide controlled release for 28 days under in vitro conditions. In order to understand the cellular entry mechanism, various endocytosis inhibitors were used to investigate the pathways of NLGs into the cell. It was observed that positively charged NLGs enter the cell through various endocytic mechanisms, assist in escaping the endosomes, and gradually deliver siRNA into the cytoplasm. Transfection studies demonstrated that the cross-linked NLG platform provided efficient gene transfer and induced gene silencing lasting up to 14 days in vitro. It was envisaged that this long-term release-enabling siRNA-NLG platform carried the potential for both basic biological research and clinical applications of siRNA-based therapies [170].

Yu and colleagues developed a chitosan–polypyrrole nanogel system camouflaged with PC-3 cell membranes to improve the effectiveness of chemotherapy and prevent metastasis. This system provided targeted treatment for advanced prostate cancer by carrying docetaxel and RANK siRNA together. Nanogels with an average size of 155.8 nm exhibited good biocompatibility, pH-sensitive release, and homology-based targeting properties. Mice in vivo studies conducted by PC-3 tumors demonstrated that the nanogel system slowed down the tumor growth and prevented bone metastasis by suppressing the RANK/RANKL pathway. This platform is promising for chemotherapy and metastasis inhibition in prostate cancer [171].

Chitosan/Pluronic hydrogels, administered by injection, have been developed for gene therapy. Acrylated chitosan and Pluronic polymers were mixed with plasmid DNA and cross-linked physically at 37 °C and then chemically with light. The degradation and DNA release rates of the hydrogel were governed by chitosan concentration and exposure period. The released DNA formed nanoparticle complexes with chitosan or Pluronic. In experiments conducted with HEK293 cells, it was shown that hydrogels containing chitosan provided higher transfection efficiency. As a result, local gene transfer might be regulated by chitosan content [172].

In a study, researchers evaluated the antitumor efficacy of chitosan-based siRNA-VEGF nanocomplexes designed to silence VEGF, a key proangiogenic mediator associated with cancer growth and spread. The nanoplexes, composed of siVEGF-A, siVEGFR-1, siVEGFR-2, and Neuropilin-1, were formulated at a 15:1 weight ratio and administered via intratumoral injection into Sprague-Dawley rats. Tumor volume was monitored for 21 days. VEGF expression was assessed by immunohistochemistry, Western blot, and reverse transcription polymerase chain reaction (RT-PCR). A 97% decrease in tumor volume was observed in the siRNA-intervened group. This effect was lower in free siRNA injection. A significant decrease in VEGF protein levels was observed, and these findings were also confirmed by RT-PCR. The results showed that chitosan/siRNA-VEGF nanoplexes significantly reduced VEGF expression and tumor volume [161].

A novel technique to develop an efficient gene carrier nanogel based on chitosan was recently introduced. It is predicated on the idea that silica, polyethylenimine, and chitosan may self-assemble via supramolecular interactions to create a stable and stiff gene delivery platform in the reverse microemulsion system. The suggested approach is straightforward and allows quick modification of the components of the final nanogel, which might enhance its gene-carrying capacity in comparison with the chemical cross-linking process. The findings demonstrated that doping the chitosan hydrogel with polyethylenimine and silica clearly improved its strength, stability, and capacity to transport genes [173].

The intracellular uptake mechanisms and distribution of folate-decorated glycol chitosan nanogels have been evaluated in order to target the folate receptor. Free folate has been shown to act via a competitive mechanism through the folate receptor by reducing uptake. Nanogel uptake occurred mainly by endocytosis due to flotillin-1 and Cdc42; siRNA suppression of these proteins reduced uptake. In addition, the actin skeleton and macropinocytosis also contributed to the process. After incubation with HeLa cells for 7 h, 50% of the nanogels distributed in endolysosomal structures and 50% in the cytoplasm. These findings indicated that glycol chitosan nanogels are suitable carriers for intracellular targeting [174].

Gene therapy by oral route requires biological strategies that will increase the efficiency of carrier nanoparticles. The researchers developed seven different types of nanoparticles based on trimethyl chitosan-cysteine (TC) and studied the effect of the protein corona formed on the surface of gene carriers delivered by mouth. In particular, TC7-type nanoparticles with high MW, medium quaternary and high sulfhydryl content interacted with the ApoB48 protein in the intestinal environment, targeting macrophages and increasing the efficiency of gene transmission. These results show that effective transport might be achieved by personalized protein corona editing in oral gene therapy [175].

Recent years have witnessed tremendous progress in the treatment of cancer, and RNA interference (RNAi)-based targeted gene silencing techniques appeared to be promising. Nevertheless, the efficient delivery of siRNA to cancer cells remained a major challenge. Currently, one of the biocompatible and biodegradable carrier systems that garnered interest for siRNA transport has been chitosan nanogels, which were derived from chitin, a nature-sourced carbohydrate polymer. The application of chitosan nanogels as siRNA carriers in cancer treatment demonstrated considerable potential and could provide a practical approach to circumvent the difficulties linked to RNAi-based treatments [176].

## 10. Genome Editing

Specific genomic sequences might be recognized and cleaved by one of the four classes of programmable nucleases used in genome editing, thereby enabling the correction of disease-causing genes. The process of genome editing makes use of two natural DNA repair systems present in cells.

Programmable nucleases include meganucleases and engineered systems including zinc finger nucleases (ZFNs), transcription activator-like effector nucleases (TALENs), and the CRISPR platform, where the first three require designing a new protein pair for each target sequence [177], which limits their versatility and scalability. In contrast, the CRISPR-Cas9 system, developed later, utilizes a single Cas9 protein guided by a specific single-guide RNA (sgRNA) that directs the complex to the target sequence via base pairing [178]. Despite its advantages, CRISPR technology faces several challenges, including immunogenicity, the risk of introducing mutations and off-target effects, the necessity of efficient delivery systems, and ethical considerations [179]. Nevertheless, the CRISPR system has been considered a promising tool for future diagnostic and therapeutic applications.

As of February 2025, over 250 clinical trials involving genome-editing nucleases have been registered, with more than 150 actively ongoing [180]. According to other sources, the total number of clinical trials involving ZFNs, TALENs, and CRISPR ranges from 85 to 105, including approximately 35 trials targeting cancer. These trials have been conducted using both ex vivo and in vivo approaches across phases I, II, and III. Delivery methods have included adeno-associated viruses, electroporation, and polymer-based carriers [181,182]. Although polymer-based delivery systems are increasingly being explored in these trials, chitosan nanogels have not yet been included in any registered clinical trials in recent years. Nonetheless, recent preclinical studies have reported significant advancements in siRNA delivery and intracellular gene regulation using chitosan-based nanogels, highlighting their translational potential.

Recent advances have focused on developing chitosan-based delivery systems for CRISPR/Cas9. For instance, Nugrahaningsih et al. [183] employed chitosan nanoparticles to deliver CRISPR/Cas9 targeting the BMP2 gene in NIH 3T3 cells. In another study, Zhang et al. [184] designed a composite chitosan nanoparticle co-loaded with paclitaxel and a CRISPR/Cas9 plasmid targeting VEGFR2 (sgVEGFR2/Cas9). This stimuli-responsive nanoplex was tested in hepatocellular carcinoma models and showed promising therapeutic effects. Li et al. [185] compared CRISPR/Cas9 with siRNA for cancer treatment. Their formulation strategy started with the preparation of trimethyl chitosan-based nanoparticles functionalized with folic acid and 2-(Diisopropylamino)ethyl methacrylate. These nanoparticles were loaded with doxorubicin and a CRISPR/Cas9 plasmid expressing sgSurvivin. Their performance was compared to similar nanoparticles containing a Survivin shRNA plasmid (iSur pDNA). The study concluded that combining chemotherapeutics with CRISPR/Cas9 offers a promising strategy for cancer therapy.

To reduce CRISPR/Cas9-associated side effects, Khademi et al. [186] developed chitosan-based CRISPR nanoparticles modified with hyaluronic acid for targeted cellular delivery and the AS1411 aptamer for nuclear targeting. Yoshinaga et al. [187] designed an oral CRISPR delivery system using phenylboronic acid-functionalized chitosan–polyethylenimine polymers aimed at targeting the mucosal barrier.

## 11. Future Perspectives and Challenges

Chitosan-based nanogels, produced through different methods and structural modification strategies combined with targeted delivery approaches, have shown potential for use in a wide variety of biological applications. In addition, they offer significant advantages compared to classical carriers due to their unique characteristics such as intracellular targeting, controlled release, and the ability to respond to environmental stimuli. However, there are some limitations such as difficulties in standardizing formulations, scalability issues, and limitations at the clinical stage. Due to the fact that chitosan is a natural biopolymer, its specifications can vary depending on the source of raw materials, MW, moisture content, and the degree of deacetylation in the production process, which is an important limitation. Therefore, the isolation source of chitosan obtained by deacetylation of chitin (such as shellfish) may also cause significant differences. This “batch-to-batch” variation causes limitations, especially in industrial-scale production and regulatory approval processes. It also complicates the reproducibility of the results obtained between different research groups, prevents the scaling of processes due to inconsistency in physicochemical properties and represents a significant obstacle in licensing processes where consistency and strict quality standards are mandatory. This is particularly problematic in gene and protein delivery applications, where even minor variations in formulation parameters can lead to significant differences in biological activity or immune responses. Moreover, manufacturing under Good Manufacturing Practices (GMP) conditions requires rigorous validation of each process step, which remains technically demanding for nanoscale systems like chitosan-based nanogels. In this context, compliance with regulatory expectations established by agencies such as the Food and Drug Administration (FDA) and European Medicines Agency (EMA) is essential. The application of advanced characterization methods and quality control processes is of great importance in order to ensure standardization. Establishing robust analytical protocols to evaluate size distribution, zeta potential, encapsulation efficiency, and release kinetics is crucial to meet regulatory standards. Additionally, the adoption of standardized production protocols and quality-by-design (QbD) strategies can contribute significantly to overcoming these translational hurdles. Furthermore, in vitro and in vivo studies might ensure that these systems are brought to the clinical field, and chitosan nanogels might be considered as critical alternatives among the new generation carrier systems. Moreover, further chemical modifications of chitosan-based systems might enhance their efficiencies and specificities in the delivery of proteins and nucleic acids. While native chitosan and its derivatives have generally demonstrated low toxicity in in vitro and short-term in vivo studies, the addition of chemical groups to the structure can increase or decrease its toxicity. However, most chitosan derivatives still maintain favorable safety profiles compared to synthetic alternatives, making them attractive candidates for drug and gene delivery. On the other hand, in vivo long-term toxicity, immunogenicity, and biodegradation kinetics remain to be elucidated before clinical translation.

One of the most important obstacles that nanosized delivery systems encounter in vivo is the formation of protein corona. Chitosan-based nanogels can interact with plasma proteins to form a “corona” layer on their surfaces, which directly affects the biodegradation, targeting, and intracellular uptake of the transporter. Therefore, the control of the surface properties of chitosan nanogels is of critical importance in terms of specific transport to the target tissue. This area represents a valuable direction for future studies aiming to optimize the biological performance of chitosan-based nanogels. Taken together, these challenges and opportunities highlight both the limitations and the great potential of chitosan-based nanogels in advanced drug delivery systems.

## 12. Summary

Overall, characteristics including biocompatibility, biodegradability, low toxicity, and modifiable structure have made chitosan-based nanogels attractive for drug delivery systems. In this review, the potential usage of chitosan nanogels for therapeutic approaches such as protein/peptide, antigen, and gene transport has been evaluated. In summary, their adaptability to carry and deliver proteins, antigens, and genetic materials positions chitosan-based nanogels as versatile candidates for a wide range of therapeutic strategies, including gene therapy, vaccine development, and protein replacement treatments.

## Data Availability

The contributions presented in this study are included in the article.

## Abbreviations

The following abbreviations are used in this manuscript:

BMP-2	Human bone morphogenetic protein 2
CH–GA	Chitosan-glycolic acid
CLM	Chitosan-based luminescent/magnetic
CMAs	Myristic acid and chitosan-based nanogels
CMC	Carboxymethyl chitosan
CMCS	Carboxymethyl chitosan
CS-g	Chitosan grafted
DD	Degree of deacetylation
DMA	2,3-dimethylmaleic anhydride
DNA	Deoxyribonucleic acid
EMA	European Medicine Agency
FDA	Food and Drug Administration
GMP	Good Manufacturing Practices
LCST	Lower critical solution temperature
MA-CMCs	Methacrylic anhydride-modified carboxymethyl chitosan
mRNAs	Messenger RNAs
MW	Molecular weight
NAR/β-CD	Naringenin–β-cyclodextrin
NG	Nanogel
NLG	Nanolipogel
OPs	Oyster peptide
PCL	Polycaprolactone
PCS	Phosphorylated chitosan
PEG	Polyethylene glycol
PNIPAAm	Poly(N-isopropylacrylamide)
PVA	Polyvinyl alcohol
QbD	Quality-by-design
recNcPDI	Nanogel-encapsulated recombinant NcPDI
RNAi	RNA interference
RT-PCR	Reverse transcription polymerase chain reaction
siRNA	Small interfering RNA
TALENs	Transcription activator-like effector nucleases
TC	Trimethyl chitosan-cysteine
TPP	Tripolyphosphate
Tyr	Tyrosine
uPA	Urokinase-type plasminogen activator
ZFNs	Zinc finger nucleases
